# Building integrated health systems: lessons from HIV, sexual and reproductive health integration

**DOI:** 10.1093/heapol/czx142

**Published:** 2017-11-24

**Authors:** Susannah H Mayhew, Jonathan Hopkins, Charlotte E Warren

**Affiliations:** 1 Department of Global Health and Development, Faculty of Public Health and Policy, London School of Hygiene & Tropical Medicine, London, UK; 2 Independent Consultant, formerly, International Planned Parenthood Federation, London, UK and; 3 Population Council, Washington, DC, USA

**Keywords:** Integration, health systems, evaluation, reproductive health, HIV, resilience

Key Messages

- Any focus on integrating health services needs to include a broader systems wide approach if it is to be successful and sustainable.
- In context of Sustainable Development Goals which recognised the interconnectedness of sectors, the ability to provide joined-up packages of services to meet changing health—and development—needs becomes more relevant.
- Health needs will change rapidly in the next 50 years with increased life expectancies, aging populations and the disease burden shift from infectious to chronic diseases and climate-related changes in vector-borne diseases. Health systems need to adapt to these changing needs.
- The integration research in this Supplement illustrates that key ways of enhancing resilience to change will be building flexible workers who support each other in teams with good communication and leadership.

Are integrated health systems more people-centred, efficient and cost-saving or even more resilient than parallel, specialist systems? Much has been claimed for ‘integrated’ approaches to primary health care but almost all research has looked at integration at the point of service delivery rather than the health system more broadly. Conversely, health systems research has not often looked at ‘integration’ models, processes or case-studies using a health systems lens.

There is surprisingly little consensus on what “integrated care” actually means with a plethora of definitions, models and consequently of measurements and assessment approaches to “integration” (Criel *et al.* 1997; Mitchell *et al.* 2004; Ekman *et al.* 2008; Atun *et al.* 2010; Dudley and Garner 2011; Lindegren *et al.* 2012). Health care delivery ranges from separately delivered, specialist programmes at primary level like in the USA (all paid through private health insurance) and former Soviet states; to the ideal of a fully integrated comprehensive primary care system as envisaged by Alma Ata. Much of the work in different income-settings has been conducted without reference to other settings, so lessons from high-income countries (HICs) have not been transferred to low-/middle-income (LMIC) settings and vice versa. Although in HICs the focus tends to be on the processes of coordinating care from different care-providers at different levels of the system for better patient outcomes (Curry and Ham 2010), in LMICs focus has been on integrating specific disease programmes, including malaria, leprosy, TB and HIV, often for reasons of efficiency and cost-effectiveness (Ekman *et al.* 2008; Atun *et al.* 2010).

It is perhaps the field of HIV with sexual and reproductive health (SRH) integration that has developed most prominence in the international literature since the discovery of HIV in the 1980s (Kennedy *et al.* 2010; Dudley and Garner 2011; Lindegren *et al.* 2012; Wilcher *et al.* 2013). There is an obvious connection between the two health issues: HIV is predominantly transmitted sexually or through pregnancy, childbirth and breastfeeding which are all reproductive health issues. It would therefore seem to make logical sense to address HIV through existing, well established, reproductive health programmes. In reality, however, huge HIV-specific funding streams were put in place to provide urgent response, which resulted in the development of separate, vertical systems to provide HIV-only services. From the mid-1990s the involvement of well organised networks of NGOs and civil society advocates to expand HIV and STI treatments, humanise care and give women back control of their bodies for childbearing (Newman *et al.* 2014; Mayhew and Colbourn 2015) created a prominence for HIV-SRH integration that other disease programmes have not matched. Since 2004 there has been increased rhetoric on the need to bring these different services, systems and related policies together (UNFPA 2004; WHO and UNFPA 2006), but still the practice of integrating systems and service delivery has proved very difficult. Over the past decade a renewed commitment to programmes and research to understand how and why integration has or has not happened provides a particularly rich field of experience to explore the health systems dimensions of integration. The single largest programme of research to date is the Integra Initiative: a longitudinal research programme to evaluate the impact of different models of integrated SRH-HIV service delivery in Kenya, Swaziland and Malawi (Warren *et al.* 2012). Some of the systems-focused results from Integra form a core part of the present Supplement together with other new studies and analyses from this rich field of research. The Supplement explores the health systems challenges, and successes, of delivering integrated services to learn the wider lessons for systems integration. We provide a collection of nine papers which include reviews, primary data studies and think pieces to bring systems processes, structures and “software” (its people) under the spotlight to inform how to achieve sustained integrated systems that can respond to the ever-changing and inter-connected health needs of diverse populations.

Mounier-Jack *et al.*’s (2017) article begins the Supplement with a commentary on the extent to which lessons can be learned between high income, and LMIC health systems vis-à-vis integrated care. During the 2000s, discussion of integrated care has become more widespread in both high and low-income settings. Yet, despite this there has been little sharing of information or learning between income settings. The authors show that, despite different contexts, there are many common features of integration across income settings as well as shared challenges in understanding, measuring and developing evidence on the results of integration. They note the challenges of providing robust evidence (in any setting) on the benefits and effects of integration, given the very wide range and heterogeneity of integrated schemes from integrated clinical care for individual patients to broader systems involving social and health care services, their complexity and the difficulties of rigorously evaluating these schemes. They therefore make a clear call for researchers from currently very separate parts of health services and systems research to start a dialogue on how to share methods and substantive knowledge to evaluate integrated care and systems comparatively in a wider range of settings and thus provide better evidence to policy-makers.

Responding to this, two systematic reviews then survey the literature from both high- and low-/middle-income settings for integration of HIV services with mental health services and with other chronic disease services respectively. Chuah *et al.* (2017) map interventions and approaches to integrating HIV and mental health services, noting the strong clinical and organizational rationales for doing this. HIV and its opportunistic infections can cause neurological damage and cognitive impairment; some forms of mental illness, including depression and associated substance use disorders, may be associated with risky behaviours that promote transmission of HIV; mental health problems can also jeopardise adherence to treatment, with major consequences for survival. There are many examples of initiatives to bring the services together in recognition of these connections, but 38 or the 45 papers reviewed came from HICs. The dearth of evidence from LMICs, particularly those with high HIV burdens, is presumably reflective of the lower attention given to mental health problems in these settings. Across the papers the authors identified three models of integration at the meso and micro levels: single-facility integration, multi-facility integration, and integrated care coordinated by a non-physician case manager. Each has their strengths and limitations but in all cases there is insufficient evidence from LMICs. Integration on a single site (or ‘one-stop shop’) has many advantages for the patient but it can be difficult and expensive to bring all the necessary services together in a single place. Multi-facility integration involves building a network between health facilities and other providers, allowing people with complex problems to obtain access to those with the specialist knowledge needed to treat them. However, here the coordination can be very difficult with the risk of fragmentation of care. The final model involves integrated care led by a case manager, with referral to specialists as needed. This also can be effective, but it requires very highly skilled case managers, who may be difficult to recruit and retain in health systems facing health worker shortages.

The second systematic review by Watt *et al.* (2017) finds more evidence from LMIC settings, though still imbalanced. The authors use an explicit health systems lens to explore what it is about health systems that either helps or hinders schemes to integrate services from achieving their full potential. They focus on integration of services for people living with HIV and those with chronic non-communicable diseases that are increasing in many LMICs (as well as HICs). Of the 150 papers reviewed, 67% were from high-income settings. The findings show that whether service delivery integration is successful depends substantially on characteristics of the health systems in which they are embedded. In particular integrated service-delivery is more likely to succeed where health systems encourage effective collaboration and coordination within and between teams, and between staff and patients. It is not just about formal systems and service structures; informal relationships and trust are equally important. Although the review confirms the importance of supportive institutional structures, dedicated resources, appropriately trained, skilled and incentivised health workers it also highlights the importance of health workers being flexible in the roles that they can perform, where necessary going beyond their core areas of work. Having a ‘go-to’ person who can act as contact point for everyone involved was also found to be helpful. They acknowledge that staff perform best when they are supported by appropriate institutional structures and dedicated resources as well as managers and leaders committed to integrating services and overcoming difficulties. Critically, they find that a positive, problem-solving culture, with a focus on the patient, their needs and personal circumstances made a difference, as did careful design of appropriate delivery models that can respond to patients’ needs, though more evidence is needed from LMICs. This often involves working with families, communities and change agents outside the health system.

Mudzengi *et al.*’s (2017) paper provides a case study of costs associated with integration of HIV services with one chronic disease, tuberculosis (TB). The case study is from the high–middle income setting of South Africa and takes up one of the critical policy issues that surround integration: cost. As in many countries HIV is a major driver of the TB epidemic in South Africa, and a major cause of death amongst TB patients. Patients with co-infections like these are at particular risk of catastrophic expenditure due to increased severity of disease, diagnostic delay, and the need for intensive health service use in settings where care is not integrated. The article describes the economic impact of TB/HIV co-infection, to identify the potential benefits of integrated care for this particularly vulnerable group. Their survey of 454 TB and HIV patients in public clinics in a sub-District of South Africa confirms that patients with TB/HIV co-infection encountered substantially higher costs than patients with TB only or HIV only. This is primarily explained by the higher number of visits required: co-infected patients on average made 27 visits over the study period, while TB only patients made an average of 20 visits, and HIV only patients made an average of 5 visits. Systems and service integration is identified as having clear potential to reduce the economic burden of co-infection, by coordinating health care appointments to reduce the number of visits necessary for patients. Work in Swaziland, where co-infection is also high, has highlighted the difficulties of coordinating appointments (Colombini *et al.* 2016) and the challenges identified in Watts *et al.*’s paper suggest that integration of these services won’t be easy. Mudzengi *et al.* (2017) also call for a response that is broader than the health sector to protect vulnerable co-infected patients with social protection and income protection schemes since their catastrophic costs amounted to an average of 33% of monthly household income.

The paper by Hopkins *et al.* (2017) moves us to the consideration of the integration of HIV and SRH policies and programmes which is the focus of the rest of the Supplement. The integration of these two related services has been a focus for service-integration initiatives for well over 20 years. Hopkins *et al.* analyse the extent to which SRH-related targets and priorities feature in the HIV strategies, and vice versa, in 60 countries. Although there is international—as well as widespread national—commitment to integrating HIV and SRH policies, and programmes, the paper confirms that most attention from donors and implementing agencies has been on service-delivery rather than health policy and systems integration. Overall, integration of SRH and HIV at the policy level remains surprisingly weak. The analysis found that HIV strategies were more likely to include related SRH priorities and targets (a global average of 6.1/10) compared to SRH strategies which largely failed to integrate related HIV priorities and targets (scoring a global average of just 3.3/10). Nevertheless some large gaps remain even within the HIV strategies referencing SRH needs. Although prevention of mother-to-child transmission is mentioned and has targets in both HIV and SRH strategies, the broader SRH needs (such as family planning) of women living with HIV are not mentioned. Also, condoms are still being seen in silos rather than as an effective triple protection tool (to prevent HIV, other sexually transmitted infections, and pregnancy). If meaningful two-way linkages are to take place and to realise the full benefits of service integration, the authors call for urgent increased effort to work with those who are developing national SRH and HIV strategic plans to ensure they are integrated with each other.

The three papers which follow are from the Integra Initiative. Integra took a service and systems perspective, using process evaluations, cost studies as well as cohort studies for impact evaluation and detailed qualitative work with clients and providers (see Warren *et al.* 2012).

The first paper, by Mayhew *et al.* (2017), seeks explicitly to explore the interactions between facility and systems structures and the people (or ‘software’) that work within them in order to explain why some facilities were able to implement and sustain integrated service delivery while other similar facilities were not. The authors draw on multiple data-sets for four facility case-studies to give a holistic perspective on the processes and perceptions of integration. The findings of their study echo some of those from the Watts *et al.* review of systems facilitators and barriers to achieving integrated service delivery and further contribute to filling the low-income evidence-gap. The case study findings show that frontline health workers and managers are able to influence how integrated care is provided even in the context of a weak health system where stock outs high provider workload and staff deployment challenges exist. Facilities where staff displayed agency of decision making, worked as a team to share workload and whose managers supported this, showed better delivery of integrated care. Moreover, staff were able to overcome some structural deficiencies to enable integrated care. Some poor-performing facilities had good structural integration, but staff were unable to utilise this because they were poorly organized, were unsupported or their teams were dysfunctional. Conscientious objection and moralistic attitudes were also barriers. Taken together, this suggests, as the Watts review hints, that structural integration is not sufficient for integrated service delivery; rather sensitive management of staff to nurture and support their agency in decision making, team-working and load-sharing is critical to being able to work flexibly to meet the challenges that face providers each day. The ability to provide such support for integrated services to build flexible, resilient health systems to meet changing needs is particularly relevant as health systems face challenges of changing burdens of disease, climate change, epidemic outbreaks and more. The need for flexibility and resilience of health systems are themes taken up again by Warren *et al.* in the final paper.

The two other Integra papers provide detailed studies of two key issues of concern to practitioners of integrated care: the impact of integrated service delivery on waiting times and on quality of care. The first paper, by Siapka *et al.* (2017), takes up the question of waiting times. This has been of particular concern for two reasons. First the concern, well-illustrated by the earlier Mudzengi paper, that having to attend two or more separate services to meet multiple health needs increases costs and time to the patient, while receiving integrated care for several health needs at the same time will reduce the patient-burden by reducing consultation times and therefore overall waiting times. Second, in resource-constrained low-income settings where skilled human resources are often lacking integration is seen as providing potential to improve service effectiveness and optimise the use of limited resources and clinical staff time. The authors present data from 24 health facilities in Kenya as part of the larger Integra Initiative to assess whether integration of provider initiated HIV counselling and testing (PITC) and FP (FP counselling and FP provision) services has an impact on consultation duration times. They compared the consultation duration times for integrated PITC and FP service consultations with those for FP-only services and PITC-only services. The findings were not entirely expected. They found that integrated PITC/FP services had longer consultation times than FP-only services, but shorter consultation times than PITC-only services. The authors note that this may be due to the fact that more pre-and post-counselling is provided at PITC-only services. The findings raise concerns about quality of HIV care since the duration of integrated PITC/FP visits fell below that required by WHO HIV testing guidelines, suggesting that while integration may reduce consultation times and therefore provider workload, it may come at the expense of quality.

Mutemwa *et al.*’s (2017) paper explores this issue of quality by looking at the technical quality of the host service (family planning). In the Siapka paper integration of PITC with FP was found to increase consultation times on average, suggesting that more time is spent with the client than would have been without the addition of HIV services. Increased consultation times for integrated services, compared to FP-only consultations, may be a good thing for family planning services but concerns persist about the impact on quality. Mutemwa’s paper uses cross-sectional data and provider interviews from 12 of the clinics in Siapka’s study. After adjusting for facility level structural factors, HIV/family planning integration was found to significantly improve technical quality of the consultation session—evidence that is new in the wider integration literature. The study looked at a range of structural and provider factors to determine whether the association between service integration and technical quality of care worked through any specific elements of the client-provider consultation session. Half of the 14 structural factors identified were positively associated (including availability of family planning commodities and reagents; adequate infrastructure and appropriate provider clinical knowledge). Five of the seven provider factors identified were positively associated (including supervision and job satisfaction) while workload was negatively associated. Technical quality of the client-provider consultation session was also determined by duration of the consultation and type of clinic visit, and appeared to depend on whether the clinic visit occurred early or later in the week. These findings further add to the review papers’ findings in the Supplement as well as Mayhew *et al.*’s paper, that both structural and people factors play a role in successful, quality integration.

Taken as a whole these primary-data papers underline the importance of a systems perspective to integration—that takes account of multiple systems elements, not merely where the services are provided together. These include what has been termed ‘systems software’ (i.e. people) who deliver (or not) integrated care and interact with the ‘systems hardware’ factors (infrastructure, training etc.) to overcome or create barriers. Findings highlight the need for health systems to support healthcare workers to promote a supportive enabling environment that can facilitate provision of integrated health services. The final perspective on integration in this Supplement is provided by Warren *et al.*’s (2017) viewpoint paper which considers the lessons from the legacy of SRH-HIV integration for wider considerations of systems integration within the context of the Sustainable Development Goals. Lessons from SRHR-HIV integration experience point to the need for strong political will to establish clear governance structures with a key role for civil society in holding governments and government agencies accountable for rights-based action on health. Another important lesson is that it is not only structures, policies and resources that must be linked but the people within the sector or system must also be motivated and enabled to make connections beyond their usual field (and sector) of operation. Like other authors in this Supplement, Warren *et al.* call for a people-centered approach and one that is cross-sectoral, noting that for policy makers, the progressive realization of the right to health depends on the development of enabling environments to support the structural linkages for planning and service-delivery across sectors which requires political will and strong leadership. Taking up the perspective of duty-bearers in a rights-based response the authors maintain that researchers have an obligation to rights holders (namely users of the services) to systematically map and analyse the connections, and the impacts of those connections, between health systems and the Sustainable Development Goals.

The articles in this Supplement clearly illustrate that any focus on integrating health services needs to include a broader systems-wide, people-centred approach if it is to be both successful and sustainable. In context of the sustainable development goals, which recognise the interconnectedness of sectors, the ability to provide joined-up packages of services to meet changing health—and development—needs becomes increasingly relevant. Moreover, health needs are going to change significantly in the coming 50 years with increased life expectancies, aging populations and the disease burden shift from infectious to chronic diseases as well as climate-related changes in patterns of vector-borne diseases. Health systems need to adapt to these changing needs and the integration research in this Supplement shows that a key way of being resilient to change and difficult conditions is building flexible workers who support each other in teams with good communication and leadership. There is a long way to go to understand how best to nurture and support such leadership and team-work in low-income settings, but it will be critical to the development of health (and other) systems that are able to meet future challenges.

## Funding

The study was funded by the Bill & Melinda Gates Foundation through the International Planned Parenthood Federation (grant number 48733). The research team is completely independent from the funders.

*Conflict of interest statement*. None declared.

## Ethics

Ethical approval was granted by the Kenya Medical Research Institute (Reference: NON/SSC/113 and 114); the Research Ethics Committee of the London School of Hygiene & Tropical Medicine (approval number 5426), and the Population Council institutional review board (approval numbers 443/444). The Integra Initiative is registered with Clinical Trials (ClinicalTrials.gov) as NCT01694862.

## Authors’ contributions

S.M. conceived the Supplement, internally reviewed all studies and drafted this Editorial. C.W. and J.D.H. both internally reviewed all studies and provided comments on this article.
